# Non-randomised evaluations of strategies to increase participant retention in randomised controlled trials: a systematic review

**DOI:** 10.1186/s13643-020-01471-x

**Published:** 2020-09-29

**Authors:** Adel Elfeky, Katie Gillies, Heidi Gardner, Cynthia Fraser, Timothy Ishaku, Shaun Treweek

**Affiliations:** 1 Health Services Research Unit, University of Aberdeen, Aberdeen, UK; 2 School of Nursing, Midwifery and Health, Coventry University, Coventry, England

**Keywords:** Randomised trials, Retention strategies, Drop-outs, Non-randomised evaluations, Participant retention

## Abstract

**Background:**

Retention of participants is essential to ensure the statistical power and internal validity of clinical trials. Poor participant retention reduces power and can bias the estimates of intervention effect. There is sparse evidence from randomised comparisons of effective strategies to retain participants in randomised trials. Currently, non-randomised evaluations of trial retention interventions embedded in host clinical trials are rejected from the Cochrane review of strategies to improve retention because it only included randomised evaluations. However, the systematic assessment of non-randomised evaluations may inform trialists’ decision-making about retention methods that have been evaluated in a trial context.Therefore, we performed a systematic review to synthesise evidence from non-randomised evaluations of retention strategies in order to supplement existing randomised trial evidence.

**Methods:**

We searched MEDLINE, EMBASE, and Cochrane CENTRAL from 2007 to October 2017. Two reviewers independently screened abstracts and full-text articles for non-randomised studies that compared two or more strategies to increase participant retention in randomised trials. The retention trials had to be nested in real ‘host’ trials ( including feasibility studies) but not hypothetical trials.

Two investigators independently rated the risk of bias of included studies using the ROBINS-I tool and determined the certainty of evidence using GRADE (Grading of Recommendations Assessment, Development and Evaluation) framework.

**Results:**

Fourteen non-randomised studies of retention were included in this review. Most retention strategies (in 10 studies) aimed to increase questionnaire response rate. Favourable strategies for increasing questionnaire response rate were telephone follow-up compared to postal questionnaire completion, online questionnaire follow-up compared to postal questionnaire, shortened version of questionnaires versus longer questionnaires, electronically transferred monetary incentives compared to cash incentives, cash compared with no incentive and reminders to non-responders (telephone or text messaging). However, each retention strategy was evaluated in a single observational study. This, together with risk of bias concerns, meant that the overall GRADE certainty was low or very low for all included studies.

**Conclusions:**

This systematic review provides low or very low certainty evidence on the effectiveness of retention strategies evaluated in non-randomised studies. Some strategies need further evaluation to provide confidence around the size and direction of the underlying effect.

## Background

Retention can be defined in several ways, for example, the ‘Standard Protocol Items: Recommendations for Interventional Trials’ (SPIRIT) guideline defines poor retention as ‘instances where participants are prematurely “off-study” (i.e., consent withdrawn or lost to follow-up) and thus outcome data cannot be obtained from them’ [1]. Retention of participants is essential to ensure the statistical power and internal validity of clinical trials. Poor retention reduces power and can bias the estimates of intervention effect, which seriously affects the credibility of trial results and the potential of a trial to influence clinical practice [2]. In a review that evaluated missing outcome data in randomised trials published in four major journals, 89% of studies reported some missing data and 18% of studies had more than 20% of participants with partly missing outcome data [3]. Recent work with a 2004–2016 cohort of trials funded by the UK Health Technology Assessment Programme found that 50% of trials did not have primary outcome data for more than 10% of participants [4].

It is generally accepted that under 5% loss to follow-up will introduce little bias, while missing outcome data from more than 20% may pose a major threat to the validity of the study [5]. Some trial results, however, can be far more vulnerable to missing data than this suggests. The Fragility Index, a way of assessing how fragile a trial conclusion is, developed by Michael Walsh and colleagues, shows that what is considered statistically significant at *P* < 0.05 can be turned insignificant by a handful of events going in the opposite direction [6]. Crucially, the same study found that for 53% of trials, the number of event swaps needed to change the conclusion was less than the number lost to follow-up. While modest missing data can be handled with statistical methods, the risk of bias can remain [7] and it is difficult to meaningfully fix substantial missing data by statistical means [8].

A Cochrane review published in 2013 on interventions to improve retention in trials identified 38 studies that evaluated retention interventions using random or quasi-random allocation [9]. The authors concluded that financial incentives increased questionnaire response rates but were unable to draw conclusions about in-person follow-up. Only four of the included studies looked at in-person follow-up and two of these evaluated strategies to improve retention to the intervention and not to the trial itself. A more recent systematic review of retention strategies for in-person follow-up in health care studies identified 88 studies, only six of which (four RCTs, one quasi-RCT and one uncontrolled trial) were designed to compare retention strategies, whereas the remainder (82 studies) described retention strategies and retention rates but offered no rigorous evaluation of strategies used [10]. The lack of included studies making direct comparisons combined with heterogeneity in the types of strategies, participants and study designs prohibited meta-analysis.

### The rationale for the review

The importance of trial retention combined with the lack of evidence regarding interventions that might improve it has led to retention being identified as one of the top three methodological research priorities in the UK [11]. Given the lack of randomised trial evidence on effective retention strategies, the contribution of evidence from non-randomised evaluations looks worthy of examination.

The potential contribution non-randomised studies can make to the evaluation of effectiveness has provoked considerable controversy [12]. Including non-randomised effect evaluations in systematic reviews could be viewed as problematic, particularly because of poor methodological quality and the likelihood of selection bias and its impact on study results. However, evidence from a recent Cochrane review of reviews has shown that there were no significant effect estimate differences between RCTs and observational studies (79% of the included reviews showed no significant differences between observational studies and RCTs) [13]. This suggests that observational studies can be conducted with sufficient rigour to provide complementary evidence or replicate the results of randomised trials. Moreover, we think that the systematic evaluation of what is expected to be a considerable amount of research is crucial; without collation, this body of evidence is currently being disregarded and may hold promising results for the trial community regardless of whether the outcomes support one or more interventions.

While accepting that non-randomised evaluations have methodological weaknesses for the evaluation of effect size, researchers nevertheless choose these designs for many studies and we believe it is worth systematically reviewing this literature to assess the usefulness of approaches evaluated using non-randomised methods, and whether they may be worth evaluating in randomised studies in the future.

## Objectives

- To provide a comprehensive review of retention strategies evaluated through non-randomised study designs.
- To measure the effect of strategies to promote retention in randomised trials and to explore whether the effect varied by trial setting, trial strategy and/or retention behaviour.

## Methods

Details of review methods used were prespecified in the published protocol [14]. This systematic review was conducted and reported in accordance with the Preferred Reporting Items for Systematic Reviews and Meta-Analyses (PRISMA) guidelines [15]. The PRISMA checklist is provided in Supplementary document (1). We briefly summarise our methods below.

### Criteria for considering studies for this review

#### Types of studies

Non-randomised studies that compared two or more strategies aimed at increasing participant retention in randomised trials. The retention trials had to be nested in real (i.e. not hypothetical) randomised ‘host’ trials, including feasibility studies. The most robust test of the effectiveness of a retention strategy is a trial comparing one retention method with an alternative, ‘nested’ within an ongoing host clinical trial. By ‘nesting’, we refer to patients being allocated to two or more alternative methods of retention by random or non-random methods. Such studies provide a context that is the same as the one we are interested in clinical trials. This makes judgements about the applicability of the evidence coming from these evaluations more straightforward than for evaluations done outside trials and/or outside healthcare. The wider experimental evidence is already described in a number of reviews, for example, Edwards et al. [16]. We also excluded randomised evaluations from our review as they were the subject of an existing Cochrane review, which is currently being updated [9].

### Outcome measures

#### Primary outcome

The primary outcome was the number of participants retained at the primary analysis point as stated in each retention study. In cases where the time points to measure the primary outcome were not predetermined, the first time point reported was considered.

#### Secondary outcomes

Retention at secondary analysis points and cost of retention strategy per participant.

### Search methods

The search strategy was constructed in discussion with an information specialist (CF) with expertise in healthcare databases and systematic reviews. The literature search was conducted using the Cochrane Methodology Register, The Cochrane Controlled Trials Register, MEDLINE and CINAHL electronic databases. The search was limited to English studies published in the last 10 years to increase relevance to current trials.

Other supplementary search methods included hand-searching of reference lists of relevant publications, included studies and systematic reviews of randomised retention strategies to identify studies that were excluded on account of being non-randomised.

Supplementary document (2) details the full MEDLINE and EMBASE search strategy, which was adapted for other databases listed above.

### Identification of eligible studies

The abstracts of all records retrieved from the search were screened by two reviewers independently (AE reviewed all studies along with either ST or HG). The full-text check was carried out for all potentially eligible studies by two review authors independently (AE and ST). Any disagreements were discussed and resolved together with a third reviewer where necessary.Where necessary, study authors were contacted to seek information to resolve any questions regarding study eligibility.

### Data extraction and management

Two reviewers (AE and TI) independently extracted information from each of the included studies using a standardised data extraction form designed for this review. Data extracted from the host trial were objective, trial setting and clinical area. Retention strategies and retention rates at different follow-up time points were extracted independently. Any disagreements were discussed and resolved.

### Quality assessment of included studies

The Cochrane ROBINS-I (“Risk Of Bias In Non-randomised Studies-of Interventions”) tool [17] was used to appraise the quality of the included studies. ROBINS-I assessment was carried out by two review authors (AE and ST). Any disagreements were discussed and resolved with a third person where necessary.

## Data synthesis

The nature of the included studies meant that much of the analysis was anticipated to be narrative. Where population, interventions and outcomes were sufficiently similar to allow for a meta-analysis, we planned to look for visual evidence of heterogeneity in forest plots and statistical evidence of heterogeneity using the chi-square test and the degree of heterogeneity quantified using the *I*^2^ statistic. However, there was a considerable heterogeneity across the interventions evaluated in these studies, even those that fell under the same intervention category rendering meta-analysis and sub-group analysis inappropriate.

Studies were analysed according to intervention type (e.g. monetary incentives, telephone interviews); interventions were grouped when their mode of delivery or content was deemed sufficiently homogeneous. To ensure the synthesis was a rigorous process, review authors (ST, KG, HG and AE) met to discuss and categorise different retention strategies from the included studies. The six broad types of strategies identified in the Cochrane review on randomised evaluations of retention interventions [9] were considered as a guiding framework before identifying new categories emerging from the included studies. Review authors reviewed different retention strategies independently and assigned each strategy to a relevant category. The individual results were then discussed, and differences were reconciled before a list of overall retention categories was finalised.

## Assessment of the overall certainty in the body of evidence

The Grading of Recommendations Assessment Development and Evaluation (GRADE) system was used to rate the certainty in the body of evidence from the included studies [18]. GRADE provides explicit criteria for rating the certainty of the evidence. It does this by rating a body of evidence as high, moderate, low, or very low certainty. The four levels of certainty provide implications for future research (the lower the quality, the more likely further research would change our confidence in the estimates, and the estimates themselves). GRADE’s approach considers five factors: risk of bias [19], imprecision [20], inconsistency [21], indirectness of the evidence to the question at hand [22], and likelihood of publication bias [23]. By convention, randomised trials start at high, non-randomised at low certainty. Concerns with any of these factors can lead to moving the rating down. Additionally, three factors—large effects, a dose-response relationship and all plausible biases would increase our confidence in the estimated effect—can lead to moving a rating upwards. As all our included studies are non-randomised, our ratings started at low, meaning that our confidence in the effect estimate is limited: the true effect may be substantially different from the estimate of the effect. GRADE assessments were applied independently by two reviewers (AE and ST). GRADE evidence profiles were created to ensure transparency of our judgements. Guidance on the use of GRADE to rate the certainty of evidence when a meta-analysis has not been performed, and instead a narrative summary of the effect was provided is still needed. GRADE is designed to be used on bodies of evidence, which also included a body of evidence comprising a single study. To make our ratings consistent when applied to a single study, we used the approach described in Treweek’s Cochrane review of interventions to increase recruitment to randomised trials [24]:

- Study limitations: downgrade all high Risk of Bias (RoB) studies by two levels; downgrade all uncertain RoB studies by one level.
- Inconsistency: assume no serious inconsistency.
- Indirectness: downgrade by one level if a proxy for actual retention is all that is presented.
- Imprecision: downgrade all single studies by one level because of the sparseness of data; downgrade further by one level if the confidence interval is wide and crosses the line where risk difference = 0.
- Reporting bias: assume no serious reporting bias.

## Results

**Fig. 1:**
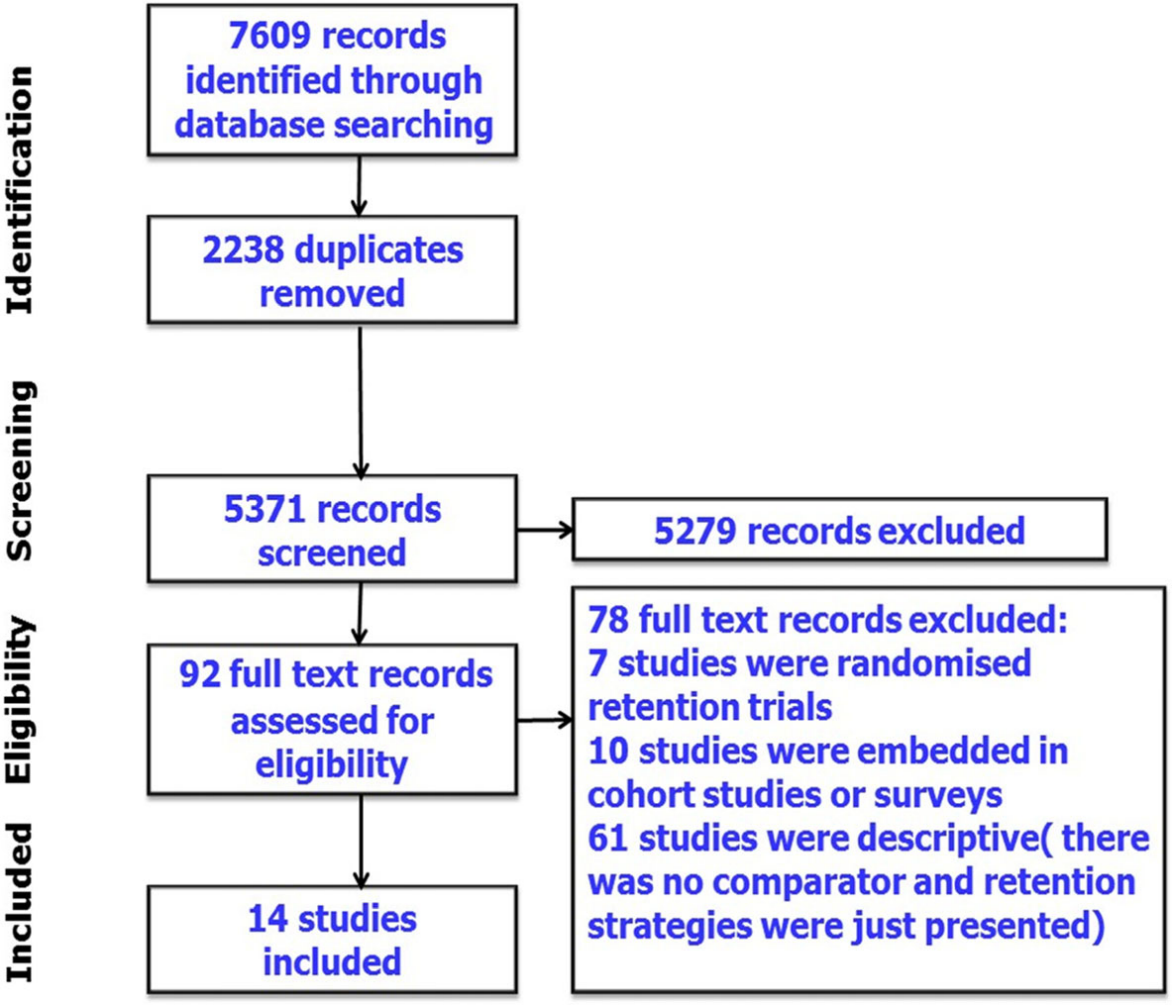
PRISMA flow diagram outlining the selection process

Seven thousand six hundred nine abstracts, titles and other records were identified, which led to 92 full-text papers, reports and manuscripts being assessed for eligiblity. Of these potentially eligible studies, 14 non-randomised retention studies were included in this review (Fig. 1).

Most retention strategies aimed to increase questionnaire response rate. The retention strategies evaluated fell into six broad categories:

1. Change in mode of data collection (e.g. from postal questionnaire completion to completion over the telephone)
2. Different questionnaire format for follow-up (e.g. short version of online questionnaire)
3. Different design strategies for follow-up (e.g. use of a run-in period to allow the participant to think further about the study and their participation, and it permits the researcher to gauge to what extent the participant will adhere to the requirements of the study)
4. Change in mode of reminder delivery (e.g. from telephone call to text messaging for follow-up)
5. Incentives (e.g. use of a monetary incentive)
6. Multifaceted strategies (e.g. intense tracing efforts to locate study participants)

## Participants and settings

Table 1 presents the characteristics of studies included in this review. Studies were conducted in a broad spectrum of clinical areas ranging from chlamydia screening to coronary artery disease and screening for traumatic brain injury. Five retention studies were UK-based, five were USA-based and the remaining four studies were set in Canada, Australia, Denmark and Malawi.

**Table 1:** Characteristics of the included studies (grouped according to the relevant retention category)

Trial	Study design	Host trial (number randomised)	Disease/condition	Participants	Setting	Retention strategies (number of participants)	Outcome (retention study)	Time point(s) used in the analysis
1. Studies that involved a change in mode of data collection								
Atherton 2010 [25]	Cohort study	Prevention of Pelvic Infection (POPI) trial (1329)	Chlamydia screening	Young female students	12 universities and colleges across London	A postal follow-up questionnaire (299) Online questionnaire (1030)	Questionnaire response rate	12 months after randomisation 4 weeks and 12 months after follow-up commenced
Childs 2015 [26]	Cohort study	Prevention of Lower Back Pain in the Military (POLM) trial (4325)	Low back pain	Geographically dispersed soldiers in the US Army	A military training setting	Web-based survey (632) Telephone call center (571) Both the web-based and telephone survey (233) M2 database (2788)	Follow-up rate	Monthly follow-up surveys (12 weeks after training). Telephone contact with soldiers who had not responded to 3 monthly web-based surveys at the end of the first year
Dormandy 2008 [27]	Before and after study	SHIFT (Screening for Haemoglobinopathies in the First trimester) (775)	Antenatal Sickle Cell and Thalassaemia (SCT) screening	People from minority ethnic groups and with high levels of material and social deprivation	UK primary care	Postal questionnaire completion only (61) Choice of telephone or postal completion (714)	Questionnaire response rate	11 months after randomisation
Johnson 2015 [28]	Before and after study	The hospital outpatient alcohol project (HOAP) (837)	Alcohol consumption	Hospital outpatients with hazardous or harmful drinking	Outpatient department of a large tertiary referral hospital in Newcastle (population 540,000), Australia	Postal questionnaire + link a web-based questionnaire (520/837) Telephone follow-up (317)	Questionnaire response rate	6 months after randomisation 4 weeks later if questionnaire remained unreturned
Lall 2012 [29]	Cohort study	Back Skills Training Trial (BeST) (701)	Back pain	Patients with subacute and chronic low back pain	UK primary care	Postal questionnaires Telephone interviews	Questionnaire response rate	12 months after randomisation 6 weeks later if questionnaire remained unreturned
Peterson 2012 [30]	Post hoc analysis method	Randomised clinical trial of elective coronary artery bypass grafting (248)	Coronary artery disease	Coronary artery bypass graft surgery patients	The New York Hospital–Cornell Medical Center	Routine follow-up approach (return to hospital for follow-up) (187) Home follow-up (61)	Follow-up rate and its impact on main trial outcome	6-month follow-up
2. Studies that used a different questionnaire format for follow-up								
Bailey 2013 [31]	Before and after study	The Sexunzipped trial (2006)	Sexual well-being	Young people aged 16 to 20 years and resident in the UK	Online study	Online questionnaire (1208) A shortened version of the online questionnaire by post (798)	Retention of valid participants at 3-month follow-up	3 months after randomisation
3. Studies that used a different design strategy for follow-up								
Ulmer 2008 [32]	Historically controlled study	Randomised controlled trial of a telephone-delivered behavioral intervention (153)	Hypertension	Participants with uncontrolled hypertension	New York Harbor Healthcare System	A 4-week simple run-in period before participation in the study	Drop-out rate	12 months after randomisation
4. Studies that used a change in mode of reminder delivery								
Hansen 2014 [33]	Cohort study	‘Preventive consultations for 20- to 40-year-old young adults’ randomised trial (495)	Change in health behavior	Young adults with multiple psychosocial and lifestyle problems	General practices in Denmark	Follow-up questionnaire and up to two reminders by mail (495) Telephone reminder to primary non-responders (179)	Questionnaire response rate	1-year follow-up after randomisation
Varner 2017 [34]	Nested cohort analysis	An RCT assessing minor traumatic brain injury (MTBI) discharge instructions (118)	Traumatic brain injury	Patients ages 18 to 64 years presenting to the ED with a chief complaint of a head injury or suspected concussion	Emergency department (ED) of an academic tertiary care hospital in Toronto, Ontario	Telephone contact (78) A reminder text message (40)	The proportional difference in loss to a follow-up between the two groups	First 4 months Final 3 months
5. Studies that used incentives								
Brealey 2007 [35]	Historically controlled study	DAMASK Trial (a pragmatic randomised trial to evaluate whether GPs should have direct access to MRI for patients with suspected internal derangement of the knee) (547)	Knee problems	Patients aged 18 to 55 with suspected internal derangement of the knee	General practices across North Wales, North East Scotland, and Yorkshire	No incentive (105) Unconditional direct payment of £5 to patients for the completion and return of questionnaires (442)	Completion rate of postal questionnaires	12 months after randomisation
Rodgers 2016 [36]	Prospective cohort study	An RCT evaluated the effectiveness of a brief social work intervention (479)	Instances of violence and heavy drinking among women	Abused women who were also heavy drinkers	Two US academic urban EDs	Cash incentives for participants enrolled during the first 8 months of the study (111) A reloadable bank card for all subsequent participants (358)	Participant completion rates of follow-up study activities and overall retention	3-, 6- and 12-month follow-up after randomisation
6. Studies that used multi-faceted strategie**s**								
Ezell 2013 [37]	Post hoc analysis method	RCT comparing asthma outcomes of students exposed to tailored asthma management versus those exposed to generic asthma management (422)	The burden of asthma	Students in grades 9 through 12	Six Detroit public high schools	Incentives ($80 for completion of all program modules) (380). 4 retention strategies to locate missing participants (re-dials of non-working telephone numbers, Facebook, assistance from school) (125)	Attrition rate	12-month follow-up
Sellers 2015 [38]	Before and after study with no control group	The BAN trial was designed to evaluate the efficacy of 3 mother-to-child HIV transmission prevention strategies (2369)	HIV prevention	Pregnant women who tested HIV-positive and their infants	Four antenatal clinics Lilongwe, Malawi	Routine strategies (support groups, home visits) (1686) Intensive tracing efforts (638)	Attrition rate	28 weeks after randomisation

### Design of the included retention studies

Twelve studies were nested in individually randomised controlled trials, and two studies were nested in cluster randomised trials [26, 27]. Five studies used before and after study design with no control group to evaluate a strategy to improve participant retention [26–28, 31, 38]. Five retention studies used a prospective cohort study design [25, 29, 33, 36, 39]. Two retention studies used a historical control study design [32, 35]. Two studies evaluated retention strategies using a post hoc analysis method [30, 37]. One retention study started after a randomised pilot study and before the main host trial [32]. All other retention studies commenced during follow-up for the host trial. All included studies targeted individual trial participants.

### Risk of bias assessment

Most (10/14) of the included studies were at low risk of bias for all ROBINS-I risk of bias domains, meaning the study is ‘comparable to a well-designed randomised study’ [17] for these domains. The exception was confounding where most of them (10/14) were at moderate risk of bias, meaning that these studies were robust for a non-randomised study with respect to this bias domain but cannot be compared to a well-conducted randomised study. Only four studies (4/14) were found to be at serious risk of bias on the confounding domain [26, 27, 32, 38]. Our judgements about risk of bias items for each and across all the included studies are presented in a risk of bias summary table (Supplementary document (3)). The risk of bias assessment was used in our GRADE judgements and in our interpretation of study findings.

### Handling missing data

The amount and reasons for missing data were recorded. Data essential to appraise the quality of included studies, numbers allocated to each group and number of participants retained at the primary endpoint were extracted. When assessing risk of bias, drop-outs were considered as a potential source of bias. The primary outcome measure for this review was retention, and this was well reported. Authors were contacted for clarification of any exclusions after randomisation to the host trial if this was unclear from retention study reports.

### Assessment of reporting bias

Although we had planned to assess reporting bias, there were too few included studies considering the same intervention to allow this to be done.

#### Intervention effects

The GRADE assessments for all comparisons are given in Supplementary document (4). One (telephone follow-up subsequent to no response to a postal questionnaire) had a GRADE assessment of low overall certainty. All other comparisons were rated as very low certainty. Results for each of the six intervention categories are given in turn. There was considerable heterogeneity across all studies, and a meta-analysis was deemed inappropriate.

#### Strategies that involved a change in mode of data collection

Six studies employed a different mode of data collection to increase retention in host randomised trials [25–30] (Table 2). Five studies were aimed at improving questionnaire response rate [25–29], and one study was aimed at reducing attrition rate and improving the accuracy of study outcome reporting [30]. Although we could not calculate a pooled effect estimate, all retention strategies evaluated seemed effective in increasing questionnaire response rates or retention ( ranging from a 14% [29] absolute increase in retention to a 41% increase [27]).

**Table 2:** Effect of strategies that involved a change in mode of data collection

Study ID	Study design	Comparator	Intervention	Difference in response rate (primary end point)	Difference in response rate (secondary end point)
Johnson 2015 [28]	Before and after study	Unique hyperlink to the follow-up questionnaire plus reminders sent at 2-week intervals (6 months after randomisation)	Telephone follow-up to non-resonders (4 weeks later)	Retention before telephone follow-up was 62.1% (520/837) and 82.8% (693/837) afterward: an increase of 20.7% (173/837)	No secondary end point reported
Childs 2015 [26]	Before and after study	3-monthly web-based surveys sent 2 years following completion of the assigned intervention	A telephone follow-up to non-responders at the end of the first year	Adding the telephone call center resulted in an 18.6% increase in follow-up rate	No secondary end point reported
Dormandy 2008 [27]	Before and after study	Postal questionnaire completion only	A choice of telephone or postal questionnaire completion	The response rate (11 months after randomisation) from women offered postal completion was 26% compared with 67% for women offered a choice of telephone or postal completion (41% difference). Response rate for women choosing telephone completion was 98% compared with 23% for women choosing postal completion (75% difference, 95% CI diff 70 to 80)	No secondary end point reported
Lall 2012 [29]	Prospective cohort study	Postal questionnaire completion 12 months after randomisation.	Telephone follow-up to nonrespondents (6 weeks later)	The overall response rate increased by 14% (from 71 to 85%) after telephone follow-up	No secondary end point reported
Atherton 2010 [25]	Prospective cohort study	Postal questionnaire completion after 12 months	Online questionnaire	The response rates to the 12-month questionnaire in the online and postal groups were 51% and 29%, respectively, 4 weeks after follow-up commenced (RR 1.78 (1.47 to 2.14))	The response rates to the 12-month questionnaire in the online and postal groups were 72% and 59%, respectively, after 3 months
Peterson 2012 [30]	Post hoc analysis method	Routine follow-up	Home follow-up	Home follow-up (6 months after randomisation) was effective in achieving follow-up on an additional 61 participants (25%), decreasing attrition rate to only 4%	No secondary end point reported

#### Strategies that used a different questionnaire format for follow-up

One study examined the effect of using a different questionnaire structure on follow-up in the context of the *sexunzipped* online randomised trial. This study examined the comparative effectiveness of a shortened version of the online questionnaire versus full version of the online questionnaire on retention of valid participants at 3-month follow-up [31]. Postal follow-up with the shortened version of the questionnaire boosted the overall response rate by 10.37% (208/2006).

#### Different design strategies for follow-up

A single study evaluated a trial design strategy as a retention intervention, the use of a 4-week period (which the authors called a run-in period) to allow participants to consider their involvement in the trial [32]. Drop-out rate decreased from 25.0% (in the pilot study) to 4.6% 12 months after randomisation in this study and retention rate increased from 75 to 95.4%.

#### Strategies that involved a change in the mode of reminder delivery

Two studies evaluated two different reminder strategies to increase response rate and decrease participant attrition [33, 34] (Table 3). Again, the specific retention strategies used in studies under this category were different and this precluded a meta-analysis.

**Table 3:** Effect of strategies that used a change in mode of reminders delivery

Study ID	Study design	Comparator	Intervention	Difference in response rate (primary end point)	Difference in response rate (secondary end point)
Hansen 2014 [33]	Prospective cohort study	A follow-up questionnaire and up to two reminders by mail	Non-responders were contacted by telephone to return postal questionnaires	Telephone contact (1 year follow-up after randomisation) raised the response by 10% from 316 (64%) to 364 (74%)	No secondary end point reported
Varner 2017 [39]	Prospective cohort study	Participants were contacted by a conventional telephone call during the 4 months of study follow-up	Non-responders (final 3 months) were sent text message reminders of upcoming telephone follow-up for the return of postal questionnaires.	Sending text messages increased response by 22% (95% CI 5.9 to 34.7%) at 2-week follow-up	Sending text messages increased response by 17.7% (95% CI − 0.8 to 33.3%) at 4-week follow-up

#### Strategies that involved incentives

Two studies evaluated the effect of offering monetary incentives to partipants to improve postal questionnaire response rates and to promote follow-up phone or in person interviews in host randomised trials [35, 36] (Table 4).

**Table 4:** Effect of incentives

Study ID	Study design	Comparator	Intervention	Difference in response rate (primary end point)	Difference in response rate (secondary end point)
Brealey 2007 [35]	Historical control study design	No incentive (the first 105 patients did not receive the £5 incentive)	The subsequent 442 patients received unconditional direct payment of £5 for the completion and return of questionnaires	The response rate (12 months after randomisation) following reminders for the historical controls was 78.1% (82 of 105) compared with 88.0% (389 of 442) for those patients who received the £5 payment (diff = 9.9%, 95% CI 2.3 to 19.1%).	No secondary end point reported
Rodgers 2016 [36]	Prospective cohort study	In-person cash incentive for the first 111 participants	The subsequent 358 participants were given reloadable bank card for incentive payments	Retention rates among the card-paid participants at 6 months was 80% vs. 68% cash-paid	Retention rates among the card-paid participants at 12 months was 72% vs. 66% cash-paid

#### Multi-faceted strategies

Two studies used multi-component strategies to trace missing study participants and increase retention [37, 38] (Table 5).

**Table 5:** Effect of multi-faceted strategies

Study ID	Study design	Comparator	Intervention	Difference in response rate (primary end point)	Difference in response rate (secondary end point)
Ezell 2013 [37]	Post-hoc analysis method	All partcipants were offered the possibility of receiving incentives ($80) for completion of all program modules and surveys)	4 retention strategies (re-dials of non-working telephone numbers, mailings to the student’s home, obtaining assistance from school administration and communication through Facebook) were used to reconnect with partcipants who were overdue for the 12-month follow-up surveys	The increase in overall questionnaire response (i.e. retention) rate was 21.6% at 12-month follow-up	No secondary end point reported
Sellers 2015 [38]	Before and after study	The first 1686 participants received routine strategies (support groups, home visits, transportation to and from study visits, frequent attempts to contact clients to reschedule missed visits)	The subsequent 683 participants received enhanced intensive tracing efforts (broadcast a radio announcement in Chichewa, the local language, hiring a community educator to trace missing participants via motorcycle)	Intensive tracing efforts increased the overall response rate from 80% to 87.8% at 28 weeks after randomisation	No secondary end point reported

#### Cost of retention strategies

Only two studies reported the costs for strategies used to retain participants [27, 35]. In the study by Brealey et al. (2007) [35], the total cost for 105 patients with no incentive was £249, and the total cost for the 442 patients with a £5 incentive was £3161. The extra cost per additional respondent was almost £50. In the study by Dormandy et al (2008) [27], the additional costs associated with telephone administration compared to postal administration were £3.90 per questionnaire for English speakers and £71.60 per questionnaire for non-English speakers.

## Discussion

### Summary of main results

This systematic review summarises recent evidence from non-randomised evaluations of strategies to increase participant retention in randomised trials. A total of 14 studies were included, evaluating six broad types of strategies to increase retention in trials by increasing questionnaire response rates. There was a considerable diversity across the interventions evaluated in these studies; even those that fell under the same intervention category were sufficiently heterogeneous to render meta-analysis and sub-group analysis inappropriate.

Strategies that led to large improvements (by more than 10%) in questionnaire response rates were telephone follow-up compared to postal questionnaire completion, online questionnaire follow-up compared to postal questionnaire, shortened version of questionnaires versus longer questionnaires, electronically transferred monetary incentives compared to cash incentives, cash compared with no incentive and reminders to non-responders (telephone or text messaging). However, each of these strategies was evaluated in just a single observational study and this led to rating down for imprecision in GRADE. The GRADE overall certainty in the body of evidence is consquently always low or very low.

Most of the included studies were at low to moderate risk of bias denoting that, for trial retention, observational studies could be conducted with sufficient rigor and that researchers’ understanding of how to handle confounding adjustments in such studies has improved in recent years. Imprecision always pulled down the overall certainty in the evidence because interventions have only been evaluated once in all cases. With replication our confidence in the effect estimates would increase; we could imagine upgrading the GRADE assessment if effects are very consistent.

### Overall completeness and applicability of evidence

Telephone calls to collect data were used in four studies as a supplementary retention method and large (from 14 to 41%) improvements in questionnaire response rate were seen in all four host randomised trials. However, administration of telephone follow-up varied among these studies with respect to the length of the trial questionnaire offered to study participants to complete over the phone and this might had an impact on questionnaire response rate as shorter questionnaires are quicker to complete compared with longer questionnaires [40].

The increase in response rate following telephone reminders (without collecting data) in the study by Hansen et al. was at the same level as in other studies, regarding both the proportion of respondents [41, 42] and the 10% increase in response rate [41, 43]. Varner et al. reported that sending reminders to study participants by text message decreased attrition rate by 22%. This is consistent with findings from three linked embedded randomised trials where text messaging was effective as a post notification reminder in increasing response rate [44]. Furthermore, Clark et al. undertook a “trial within a trial” of using electronic prompts (SMS and email) to increase response rates within a randomised trial of COPD diagnostic screening. Electronic prompts increased the overall response rates by 8.8%. The results from this study were pooled in a meta-analysis with another two trials identified from Brueton’s Cochrane review. The difference in response rates was found to be 7.1% (95% CI 0.8%, 13.3%) [45].

In one study [35], the direct payment of £5 significantly increased the completion of postal questionnaires at negligible increase in cost. Brueton’s Cochrane review identified that incentives may increase the number of questionnaires returned per 1000 participants, but has only been tested in online questionnaires [9]. The use of wireless incentives provided via generic reloadable bank cards increased participant completion rates of follow-up study activities and overall retention of women drinkers in abusive relationships in a large, randomised, clinical intervention trial [36]. In this study, wireless payment more than tripled (from 27 to 97%) the number of participants who chose to complete follow-up interviews by phone, as opposed to returning to the ED for in-person follow-up interviews. This supports that a reloadable participant incentive system that does not require participants to return to the study site allows for greater flexibility of collecting follow-up data, particularly when paired with remote data collection methods. Again, this is only based on the results of one study and this stratgey needs further evaluation to determine its effect.

The shortened version of a questionnaire was used in one study, and a large improvement in questionnaire response rate was seen in the host randomised trial when compared to using the long version. This effect is consistent with the randomised trial evidence from a systematic review and a meta-analysis of 38 randomised trials evaluating the effect of questionnaire length on response rates [40]. Where participants are well and engaged with a trial, questionnaire length might not impact on response rates because trial participants may be happy to feedback on their condition in this way. For other conditions where participants’ symptoms are problematic, for example, cancers, participants may prefer shorter questionnaires.

The evidence was less clear whether multi-faceted retention strategies (i.e. several strategies used together) increased response. Several methods may be necessary for optimal retention, but it was unclear which strategy might be linked with successful contact with non-responders.

Only one study from a low-income country was identified. Accordingly, the retention strategies identified by this review may not be generalisable to trials conducted in low-income countries because the interventions identified might not be culturally, socially or economically appropriate for trials based in these regions. The applicability of the results to all social groups may be questionned as response/retention was not examined by social characteristics such as social class and economic disadvantage.

### Limitations

Several limitations of our study should be acknowledged. Although broad search terms were used, and reference lists were hand-searched, we may not have identified all publications. We are confident that we have captured most studies and the spectrum of strategies that have been evaluated in observational studies to date. It is conceivable, however, that ongoing or unpublished studies might have been missed. Although most of the retention studies were fairly well conducted and accounted for confounding factors, some were often poorly reported. Due to the considerable limitations of the evidence identified using GRADE, it was not possible to make meaningful and robust conclusions about the relative effectiveness of different retention strategies evaluated in observational studies included in this review.

### Implication for methodological research

Over the years, research conducted to change the global landscape of how retention problems in trials could be tackled has not substantively reduced our uncertainty with regards to which interventions make retention more likely. The chief reason behind this is a preference for methodology researchers to evaluate new interventions rather than to replicate evaluations of existing interventions. One way to fill gaps in the evidence is to run several Studies Within A Trial, or SWATs, a self-contained research study that is embedded within a host trial with the aim of evaluating or exploring alternative ways of delivering or organising a particular trial process. All the included studies in our review can be considered to be SWATs. In future, rather than reporting what has been done retrospectively, we would encourage trialists to prospectively plan to embed retention strategies, specifically using a SWAT protocol, into their trials from the very beginning of the process of planning the host trial. Many retention strategies used by trialists in practice were not eligible for the Cochrane review of randomised evaluations of retention interventions but were evaluated in studies within our review (e.g. home visits, telephone interviews and the use of a 4-week reflection period). Some of these interventions were linked to large improvements in retention and could be replicated in randomised SWATs to increase certainty in the evidence of their effectiveness. Telephone interviews, for example, were used in four studies as a supplementary retention method, and large (14–41%) improvements in questionnaire response rate were seen in all four host randomised trials. Although meta-analysis was deemed inappropriate, effect sizes in these studies were large enough to suggest that more rigorous evaluations are worth doing and would improve the evidence base for this intervention by confirming (or refuting) observational evidence. Moreover, offering multiple approaches to collect data such as home visits or telephone interviews were among the top five recommended practices to mitigate missing data recommended by chief investigators from 10 trials (20%) in a recent survey of 75 chief investigators of NIHR Health Technology Assessment (HTA)-funded trials starting between 2009 and 2012 [46]. Having rigorous evidence behind this recommendation would be reassuring. Treweek and colleagues have published a Trial Forge guidance document on how to design and run SWATs [47], and in the UK, the National Institute for Health Research (NIHR) has launched a funding scheme for SWATs in the Health Technology Assessment (HTA) program. The Health Research Board in Ireland runs a similar scheme. Queen’s University Belfast in Northern Ireland hosts a SWAT repository (go.qub.ac.uk/SWAT-SWAR), which contains a list of prepared SWAT protocols.

Based on the results of this review, we suggest a list of retention interventions that warrant further testing, ideally through randomised evaluations:

- The effect of telephone interviews versus online questionnaire completion on questionnaire response rate.
- The effect of home follow-up versus routine follow-up on retention rate.

## Supplementary information

**Additional file 1.** Supplementary document (1-4).

## Abbreviations

EMBASE: Excerpta Medica dataBASE
MEDLINE: Medical Literature Analysis and Retrieval System Online
RCT: Randomised control trial
PRISMA-P: Preferred Reporting Items for Systematic Review and Meta-Analysis Protocols
MeSH: Medical Subject Heading
ROBINS-I: Risk Of Bias In Non-randomised Studies-of Interventions
OR: Odds Ratio
GRADE: Grading of Recommendations Assessment, Development and Evaluation
COPD: Chronic Obstructive Pulmonary Disease
ED: Emergency Department
