# Supplementary material for: Non-randomised evaluations of strategies to increase participant retention in randomised controlled trials: a systematic review
Source: Syst Rev. 2020 Sep 29;9:224. doi: 10.1186/s13643-020-01471-x (PMC7523052; doi:10.1186/s13643-020-01471-x)
Supplement: Supplementary file 1 — Additional file 1. Supplementary document (1-4). [file 13643_2020_1471_MOESM1_ESM.docx]

**SUPPLEMENTARY DOCUMENT (1) PRISMA CHECKLIST**

| **Section/topic** | **#** | **Checklist item** | **Reported on page #** |
| --- | --- | --- | --- |
| **TITLE** | | |  |
| Title | 1 | Identify the report as a systematic review, meta-analysis, or both. | 1 |
| **ABSTRACT** | | |  |
| Structured summary | 2 | Provide a structured summary including, as applicable: background; objectives; data sources; study eligibility criteria, participants, and interventions; study appraisal and synthesis methods; results; limitations; conclusions and implications of key findings; systematic review registration number. | 3,4 |
| **INTRODUCTION** | | |  |
| Rationale | 3 | Describe the rationale for the review in the context of what is already known. | 6 |
| Objectives | 4 | Provide an explicit statement of questions being addressed with reference to participants, interventions, comparisons, outcomes, and study design (PICOS). | 7 |
| **METHODS** | | |  |
| Protocol and registration | 5 | Indicate if a review protocol exists, if and where it can be accessed (e.g., Web address), and, if available, provide registration information including registration number. | 7 |
| Eligibility criteria | 6 | Specify study characteristics (e.g., PICOS, length of follow-up) and report characteristics (e.g., years considered, language, publication status) used as criteria for eligibility, giving rationale. | 7,8 |
| Information sources | 7 | Describe all information sources (e.g., databases with dates of coverage, contact with study authors to identify additional studies) in the search and date last searched. | 8,9 |
| Search | 8 | Present full electronic search strategy for at least one database, including any limits used, such that it could be repeated. | 51,52 |
| Study selection | 9 | State the process for selecting studies (i.e., screening, eligibility, included in systematic review, and, if applicable, included in the meta-analysis). | 9 |
| Data collection process | 10 | Describe method of data extraction from reports (e.g., piloted forms, independently, in duplicate) and any processes for obtaining and confirming data from investigators. | 9 |
| Data items | 11 | List and define all variables for which data were sought (e.g., PICOS, funding sources) and any assumptions and simplifications made. |  |
| Risk of bias in individual studies | 12 | Describe methods used for assessing risk of bias of individual studies (including specification of whether this was done at the study or outcome level), and how this information is to be used in any data synthesis. | 9,10 |
| Summary measures | 13 | State the principal summary measures (e.g., risk ratio, difference in means). |  |
| Synthesis of results | 14 | Describe the methods of handling data and combining results of studies, if done, including measures of consistency (e.g., I^2^) for each meta-analysis. | 10 |

| **Section/topic** | **#** | **Checklist item** | **Reported on page #** |
| --- | --- | --- | --- |
| Risk of bias across studies | 15 | Specify any assessment of risk of bias that may affect the cumulative evidence (e.g., publication bias, selective reporting within studies). | 22,23 |
| Additional analyses | 16 | Describe methods of additional analyses (e.g., sensitivity or subgroup analyses, meta-regression), if done, indicating which were pre-specified. |  |
| **RESULTS** | | |  |
| Study selection | 17 | Give numbers of studies screened, assessed for eligibility, and included in the review, with reasons for exclusions at each stage, ideally with a flow diagram. | 12 |
| Study characteristics | 18 | For each study, present characteristics for which data were extracted (e.g., study size, PICOS, follow-up period) and provide the citations. | 15-21 |
| Risk of bias within studies | 19 | Present data on risk of bias of each study and, if available, any outcome level assessment (see item 12). | 22 |
| Results of individual studies | 20 | For all outcomes considered (benefits or harms), present, for each study: (a) simple summary data for each intervention group (b) effect estimates and confidence intervals, ideally with a forest plot. | 23-31 |
| Synthesis of results | 21 | Present results of each meta-analysis done, including confidence intervals and measures of consistency. |  |
| Risk of bias across studies | 22 | Present results of any assessment of risk of bias across studies. | 22,54 |
| Additional analysis | 23 | Give results of additional analyses, if done (e.g., sensitivity or subgroup analyses, meta-regression. |  |
| **DISCUSSION** | | |  |
| Summary of evidence | 24 | Summarize the main findings including the strength of evidence for each main outcome; consider their relevance to key groups (e.g., healthcare providers, users, and policy makers). | 32-34 |
| Limitations | 25 | Discuss limitations at study and outcome level (e.g., risk of bias), and at review-level (e.g., incomplete retrieval of identified research, reporting bias). | 34 |
| Conclusions | 26 | Provide a general interpretation of the results in the context of other evidence, and implications for future research. | 35-36 |
| **FUNDING** | | |  |
| Funding | 27 | Describe sources of funding for the systematic review and other support (e.g., supply of data); role of funders for the systematic review. | 37 |

*From:*  Moher D, Liberati A, Tetzlaff J, Altman DG, The PRISMA Group (2009). Preferred Reporting Items for Systematic Reviews and Meta-Analyses: The PRISMA Statement. PLoS Med 6(6): e1000097. doi:10.1371/journal.pmed1000097

For more information, visit: **www.prisma-statement.org**.

**SUPPLEMENTARY DOCUMENT (2): MULTIFILE SEARCH STRATEGY FOR MEDLINE AND EMBASE**

1 (attrition adj2 (minimi$ or prevent$ or lessen$ or decreas$ or reduc$)).tw.

2 (drop$-out$ adj2 (minimi$ or prevent$ or lessen$ or decreas$ or reduc$)).tw.

3 (dropout$ adj2 (minimi$ or prevent$ or lessen$ or decreas$ or reduc$)).tw.

4 (strateg$ adj2 (dropout$ or drop$-out$)).tw.

5 ((lost or loss) adj2 (follow-up or followup)).tw.

6 (withdrawl$ adj2 (minimi$ or prevent$ or lessen$ or decreas$ or reduc$)).tw.

7 (strateg$ adj2 (attrition or followup or follow-up)).tw.

8 (retention adj5 (increas$ or encourag$ or maximi$ or promot$ or improv$ or influenc$ or success$)).tw.

9 (compliance adj2 (increas$ or encourag$ or maximi$ or promot$ or improv$)).tw.

10 (strateg$ adj2 response$).tw.

11 (questionnaire$ adj3 respon?e$ adj2 (strateg$ or increas$ or encourag$ or maximi$ or promot$ or improv$)).tw.

12 (retention adj1 rate$).tw.

13 (attrition adj1 rate$).tw.

14 (follow up adj1 rate$).tw.

15 (retention adj3 (strateg$ or intervention? or method$ or technique$)).tw.

16 (compliance adj3 (strateg$ or intervention? or method$ or technique$)).tw.

17 (questionnaire$ adj3 response$ adj2 (method$ or technique$)).tw.

18 ((incentive$ or reminder$ or method$) adj3 (Retention or respon?e$)).tw.

19 (difficult$ adj2 (retain$ or retention)).tw.

20 (retention adj3 (participant? or subject? or patient?)).tw.

21 ((increase or maintain$) adj3 (partipa$ or respon?e$ or compliance)).tw.

22 Patient Dropouts/ use ppez

23 Patient Dropout/ use emef

24 or/1-23

25 research subjects/ use ppez

26 research subject/ use emef

27 exp Clinical Trials as Topic/ use ppez

28 exp "clinical trial (topic)"/ use emef

29 Observational Study as Topic/

30 ((research or trial? or study or studies or pilot or program$ or longitudinal or prospective or retrospective) and (attrition or drop$ out$ or dropouts or withdrawl$ or follow up or retention or retain$ or compliance or participation or recruit$ or engag$)).ti.

31 or/25-30

32 24 and 31

33 (letter or editorial or comment or note or abstract).pt.

34 32 not 33

35 limit 34 to english language

36 limit 35 to yr="2010 - 2017" [Embase 3890 MEDLINE 3085]

37 limit 36 to yr="2010 - 2014"

38 remove duplicates from 37

39 limit 36 to yr="2015 - 2017"

40 remove duplicates from 39

41 38 or 40

supplementary document (3) : Risk of bias summary: Review authors' judgements about each risk of bias item

|  | Atherton 2010 | Peterson 2012 | Ulmer 2008 | Johnson 2015 | Hansen 2014 | Brealey 2007 | Childs 2015 | Varner 2017 | Dormandy 2008 | Sellers 2015 | Rodgers 2016 | Ezell 2013 | Lall 2012 |   |
| --- | --- | --- | --- | --- | --- | --- | --- | --- | --- | --- | --- | --- | --- | --- |
| Baseline and time-varying confounding | Moderate | Moderate | Serious | Moderate | Moderate | Moderate | Serious | Moderate | Serious | Serious | Moderate | Moderate | Moderate | Moderate |
| Bias in selection of participants into the study | Low | Low | Low | Low | Low | Low | Low | Low | Low | Low | Low | Low | Low | Low |
| Bias in classification of interventions | Low | Low | Low | Low | Low | Low | Low | Low | Low | Low | Low | Low | Low | Low |
| Bias due to departure from intended interventions | Low | Low | Low | Low | Low | Low | Low | Low | Low | Low | Low | Low | Low | Low |
| Bias due to missing data | Low | Low | Low | Low | Low | Low | Low | Low | Low | Low | Low | Low | Low | Low |
| Bias in measurement of outcomes | Low | Low | Low | Low | Low | Low | Low | Low | Low | Low | Low | Low | Low | Low |
| Bias in selection of the reported result | Low | Low | Low | Low | Low | Low | Low | Low | Low | Low | Low | Low | Low | Low |
| Overall risk of bias | Moderate | Moderate | Serious | Moderate | Moderate | Moderate | Serious | Moderate | Serious | Serious | Moderate | Moderate | Moderate | Moderate |

supplementary document (4): GRADE evidence profile to assess the certainty in the body of evidence

| **Retention category** | **Contributing studies** | **Risk of bias** | **Inconsistency** | **Indirectness** | **Imprecision** | **Publication bias** | **Certainty(overall score)** |
| --- | --- | --- | --- | --- | --- | --- | --- |
| Strategies that involved a change in mode of data collection | (25-30) | Moderate risk (only 2 studies with serious risk of bias) | No serious inconsistency | Not serious | Serious | Undetected | **Low** 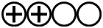 |
| Strategies that involved offering incentives to study participants | (35,36) | Not serious | No serious inconsistency | Not serious | Serious | Undetected | **Very low**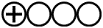 |
| Strategies that used reminders to improve retention | (33,39) | Not serious | No serious inconsistency | Not serious | Serious | Undetected | **Very low**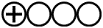 |
| Design strategies | (32) | Serious | No serious inconsistency | Not serious | Serious | Undetected | **Very low**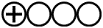 |
| Strategies that used a different questionnaire structure | (31) | Not serious | No serious inconsistency | Not serious | Serious | Undetected | **Very low**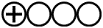 |
| Multifaceted retention strategies | (37,38) | Moderate risk (one study with serious risk of bias | No serious inconsistency | Not serious | Serious | Undetected | **Very low**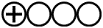 |


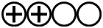
 **Low** = This research provides some indication of the likely effect. However, the likelihood that it will be substantially different** is high.


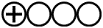
 **Very low** = This research does not provide a reliable indication of the likely effect. The likelihood that the effect will be substantially different** is very high
